# Supplementary material for: Targeting Hypoxia Sensitizes TNBC to Cisplatin and Promotes Inhibition of Both Bulk and Cancer Stem Cells
Source: Int J Mol Sci. 2020 Aug 12;21(16):5788. doi: 10.3390/ijms21165788 (PMC7461107; doi:10.3390/ijms21165788)
Supplement: Supplementary file 1 [file ijms-21-05788-s001.pdf]

Supplemental Figure 1

A2780 Ovarian Cancer Cells

Min Max

	Cisplatin Sensitive					Cisplatin Resistant					
	1	2	3	4	5	1	2	3	4	5	
**A EGFR**											
INSIG1	1100	1800	1950	1800	1400	4800	4250	6050	6400	4150	***
STAT3	2806.1	3939.8	3752.6	4145.1	2820.3	6082.6	5729.9	5634.8	3895.3	4919.7	*
EPS15L1	601.5	569.2	808.2	815	688	885.4	970.8	1143.3	1036.6	1338	**
AKT1	1600	3000	2900	3700	1400	2900	4100	3800	4800	4750	*
**B Hypoxia**											
HIF1A	33401.9	36144.6	35288.1	27652.9	29332.6	45432.6	45365.2	43787.2	37478.7	35365	***
**C Stemness**											
ALDH7A1	6200	6000	6000	8500	7500	9500	8800	9000	10500	11000	***
ALDH9A1	8000	9900	12000	10000	9000	14500	14400	14400	17000	15000	**
ALDH3B2	7.9	8.8	87.3	15.8	30	74.2	68.8	106.9	104.8	48.9	*
**D Apoptosis**											
BIK	275	356.7	82.9	368.8	416.7	14.9	157.6	119.1	147.3	48.5	*
CASP12	518.3	274.8	131.1	319.5	338.3	95.9	30.9	5.8	71.7	77.6	**
CASP9	1398.3	1531.2	1420.7	1585.6	1267.9	1197.8	1180.2	1019.5	1112.1	1127	**

Supplemental Figure 2

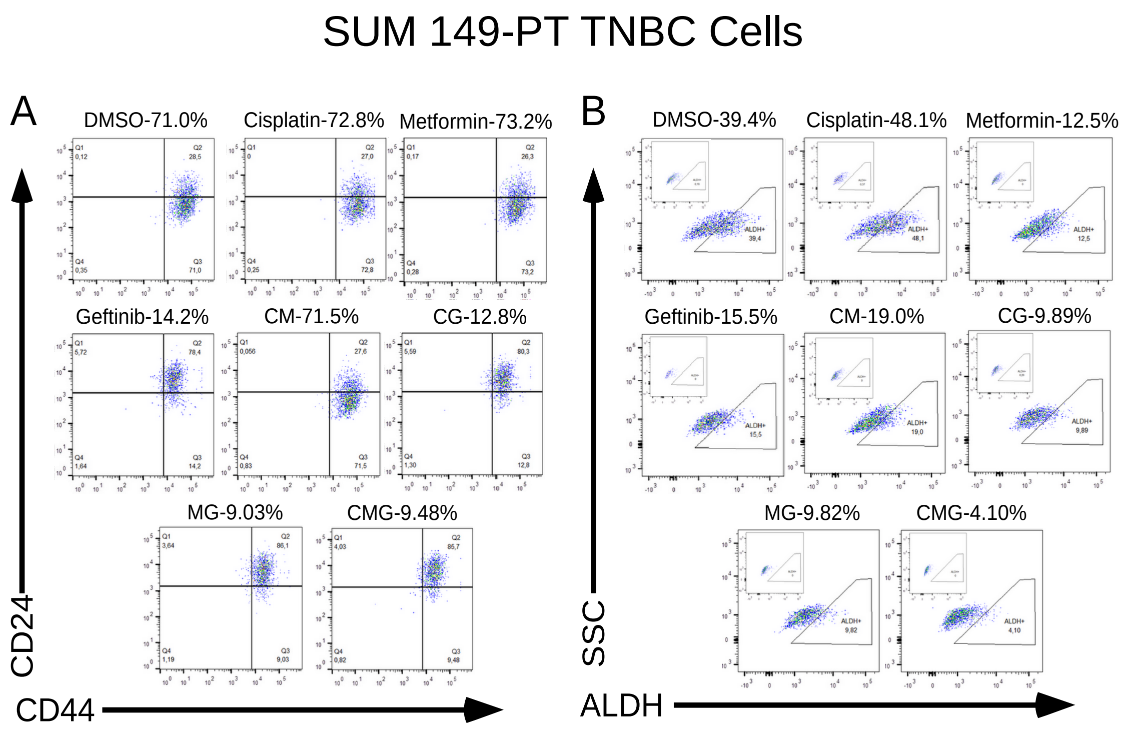

Supplemental Figure 3

TNBC Patient Tumors vs. TNBC Cell Lines

A

EGFR

EGFR $p=1.07 \times 10^{-10}$

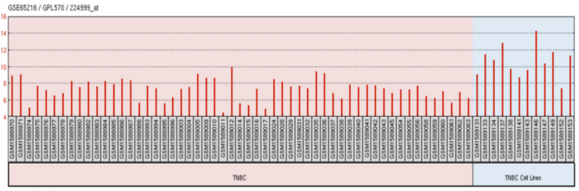

MAP2K7 $p=3.48 \times 10^{-13}$

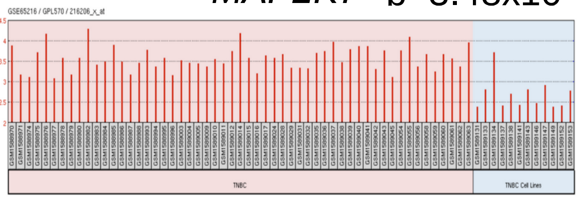

B

Apoptosis

MCL1 $p=2.95 \times 10^{-05}$

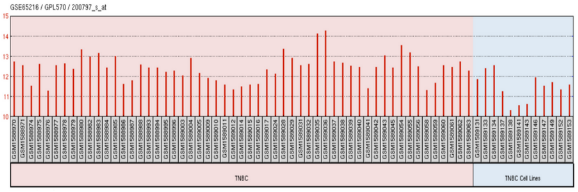

C

Hypoxia

HIF1A $p=5.51 \times 10^{-07}$

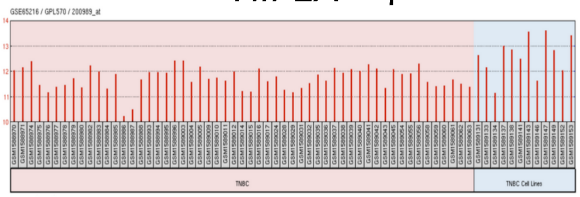

VEGFA $p=1.69 \times 10^{-02}$

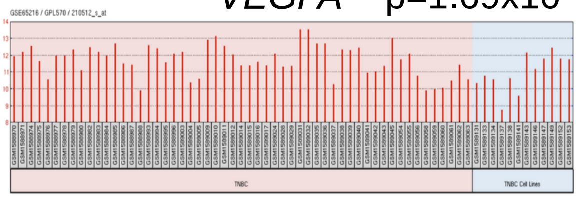

ALDH2 $p=2.09 \times 10^{-05}$

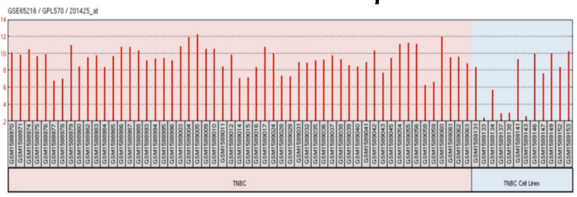

Figure Legends

Supplemental Figure 1. Cisplatin resistance is associated with high expressions of hypoxia, EGFR, stemness and anti-apoptosis related genes in ovarian cancer. (A-D) The relative expression levels (A.U arbitrary unit) of genes in 5 cisplatin sensitive and 5 cisplatin resistant ovarian cancer samples. A2780 ovarian cancer samples were compared using the NCBI Gene Expression Omnibus (GEO2R). The GSE15709 samples were analyzed using the GPL570 Affymetrix Human Genome U133 Plus 2.0 Array and the values were profiled in a heat map with green representing the row minimum and red representing the row maximum per gene analysed.

Supplemental Figure 2. Co-inhibition of hypoxia and EGFR in combination with cisplatin effectively suppress CSC populations in SUM 149-PT TNBC cells. (A-B) Representative flow cytometric plots of the CSC subpopulations (CD44 + /CD24− or ALDH+) in SUM 149-PT cells after 120 hours of treatment with the drugs in different combination.

Supplemental Figure 3. Differential gene expression in patient TNBC *vs.* TNBC cell lines. (A-C) The relative expression levels (A.U arbitrary unit) of genes in 55 TNBC patient samples and 12 TNBC cell lines using the NCBI Gene Expression Omnibus (GEO2R). GSE65216 samples were analyzed using the GPL570 Affymetrix Human Genome U133 Plus 2.0 Array.
